# 10th International Multithematic Scientific Bio-Medical Congress (IMBMC), Nicosia, Cyprus, 2022

**DOI:** 10.1038/s41419-023-05739-7

**Published:** 2023-03-29

**Authors:** Panayiota Christodoulou, Maria-Areti Salamouri, Ioannis Papavasileiou, Theodora-Christina Kyriakou, Anastasis Stephanou, Petros Agathaggelou, Ioannis Patrikios

**Affiliations:** 1 School of Medicine, European University Cyprus, Nicosia, Cyprus; 2 School of Dentistry, European University Cyprus, Nicosia, Cyprus

**Keywords:** Epidemiology, Experimental models of disease

The 10th International Multithematic Bio-Medical Congress (IMBMC) 2022, “Bio-Medical Scientific Cyprus,” took place at European University Cyprus (EUC), Nicosia, Cyprus, under the auspices of the Ministry of Health and the Cyprus Medical Association. IMBMC is an internationally recognized event that was founded and established by Professor Dr Ioannis Patrikios, the Deputy Dean and Faculty member of the School of Medicine at EUC. During the 10th IMBMC, both Sir Gregory Winter (Nobel Prize in Chemistry, 2018, on protein and antibody engineering and antibody therapies) and Sir Martin Evans (awarded the 2007 Nobel Prize in Physiology or Medicine for his groundbreaking discoveries on embryonic stem cells and DNA recombination in mammals) were announced as Honorary Professors of the School of Medicine, European University Cyprus.

The first honorary keynote speaker Professor Sir Gregory Winter’s contribution engaged in the science of protein engineering. Being the founder of both Cambridge Antibody Technology (1989) and Domantis (2000), he spearheaded the use of a new class of drugs using engineered antibody technology to treat pathological diseases. At the Laboratory of Molecular Biology, Dr Winter focused on innovating techniques to familiarize the use of antibodies in the field of therapeutics, with his goal being to develop entirely humanized antibodies, using combinational gene repertoires. Over half of the antibodies sold today are a result of his inventions, including the humanized antibodies Campath-1H, Herceptin, Avastin, Synagis, and the first human antibody (Humira) to receive approval by the US Food and Drug Administration. Precisely, antibodies, as today’s principal biological drug, especially for the treatment of cancer and autoimmune diseases, have replaced areas that were poorly served by chemical drugs. Will such developments thrive.

Professor Dr Kypros Herodotou Nicolaides spoke on preeclampsia (PE), which is a leading cause of maternal mortality and severe morbidity, in association with increased perinatal risks. He focused on ways to approach PE. These included actions on specific weeks and interventions to tackle PE, the rate, and the rate of term. Three methods were used. The first method to target PE was aspirin (150 mg per day from 12 to 36 weeks reduced the rate of PE <32 weeks by 90%, PE <37 weeks by 60%), which had no effect on term PE. The second one included the incorporation of maternal characteristics in blood pressure, serum placental growth factor, and serum sFlt-1, which identified about 70% of women who developed term PE. Unfortunately, it was found that the use of pravastatin did not reduce the rate of term PE. The last approach to prevent term PE involved screening at 36 weeks with planned delivery at 37, 38, 39, and 40 weeks, respectively. This method was considered to reduce the rate of PE by more than 50%.

Professor Dr Gregg L. Semenza gave a speech that featured his lab discovery of hypoxia-inducible factor 1 (HIF-1). As he mentioned, a continuous supply of oxygen is necessary for each of the fifty trillion cells in the adult human body. HIF regulates thousands of genes according to oxygen availability, a discovery that awarded him with the 2019 Nobel Prize in Physiology or Medicine. The aim targeted the inhibition of cancer progression, relying on the molecular mechanisms of oxygen homeostasis, in association with HIF-1. It was intended to develop HIF inhibitors that could treat cancer and blinding eye diseases.

Professor Dr Stylianos E. Antonarakis presented two research themes, the former entitled “Human Genomes and the Evolution of Medicine” and the latter “How to make an external ear: the story of FOXI3.” The first study focused on the human genome sequence and variation as a fundamental component in health and disease. He stated the importance and impact of genomic variation on phenotypic variation and the evolving knowledge of individual genomic variation; the practice of medicine is gradually evolving. Diagnosis, prevention, and therapy are all evolving as the mysteries of the genome are elucidated. Genomic Medicine takes the spotlight regarding the etiology of the myriad of constitutional and somatic disorders and raises expectations for the development of rationalistic and intelligent therapeutic methods. His second speech focused on the developmental disorder craniofacial microsomia (CFM), which has a variety of manifestations, including external ear deformity. The CFM inheritance structure is obscure and debatable. The researchers identified pathogenic variants in the transcription factor FOXI3 that cause one form of CFM. Observations in human and mouse studies point to a recessive mode of inheritance in which the phenotypic diversity is caused by a fusion of rare (causative) and common (modifier) FOXI3 alleles. His studies pertain to the relationship between genomic variability and phenotypic variation that he has studied throughout his life.

Professor Dr Philippe Menasché, a cardiac surgeon, gave a presentation entitled “Cells for Heart Failure: Replacement Therapy or Paracrine Signaling?” He noticed the benefit in terms of function even though the cells were no longer physically present in the transplanted tissue, which prompted a change from the original idea of replacement therapy to paracrine signaling, in which the combination of biomolecules secreted by the cells and primarily gathered in extracellular vesicles (EV) harness endogenous repair pathways. Despite the issues presented in the field, biodistribution and fate-tracking studies suggest that intravenously delivered cells or their secreted products are trapped in remote organs with very limited cardiac homing even though using EV from cardiac-committed cells may improve their targeting at same-tissue recipient cells. The bridge between this remote sequestration and a cardiac benefit might be a shift of the phenotype of locally present endogenous immune cells toward a reparative pattern. Thereby making these cells the conveyors of the cell- or secretome-induced protective effects. Thus, while the initial hypothesis underlying the use of cells for treating heart failure was that they could act as a replacement therapy, the current trend is to rather consider them as inducers of paracrine signaling. In the case of heart failure, but also for other conditions, the major effect of this signaling seems to be a modulation of systemic inflammation whose benefits then translate at the level of the diseased organ. More recently, the group has refocused its interest toward leveraging the paracrine effect of cells to generate a cellular secretome, which might help streamline clinical applications.

Professor Dr Paul Moss, who specializes in the field of hematology, presented: “From Diagnostics to Therapeutics; Antibodies Take Centre Stage in COVID-19,” focusing on the worldwide increase in mortality rates due to COVID-19. He highlighted that both innate and adaptive immune systems provide partial protection against reinfection of the disease. Spike-specific antibodies are the major “correlate of protection” following vaccination, and individual responses depend on a range of factors such as age, gender, and comorbidity as specifically heighted. Considering that the coronavirus distinguishes among other infections, as the biological basis is unclear and further studies should be conducted around memory B cells and plasma cells, antibodies have also emerged as powerful therapeutic agents. Hence, antibodies have been the spotlight in the control, prevention, and policy management of the COVID-19 pandemic. The information that has been derived from this challenge can now be applied effectively to a range of other medical conditions.

Professor Dr Nikolai N. Korpan specializes in cryosurgery, which is defined as clinical implications that are used at extremely low temperatures, including an organ preservation technique. He presented his unique longstanding clinical experiences with ultra-low temperatures in treating patients with severe primary and secondary malignant diseases worldwide. Ice crystallization processes are of high importance, which damage the protein denaturation and rupture the cell membranes by the action of subzero cold in intracellular ice formation. This anti-cancer concept includes radical and palliative cryosurgical operations. Cryosurgical palliative methods with a pain reduction (painlessness or pain reduction) and fetor ex ore as well as improvement of the general state by getting the tumor under control are to achieve the major subjective facilitation with cancer patients, as he noted. Hence, in the near future, a new norm for oncological diagnosis and surgery will set a new bar for modern science and modern medicine. These theoretical stages will soon become a reality in medical practice according to his personal estimation.

Professor Dr Paolo Madeddu discussed the topic entitled “Using Pericytes to Mend Broken Hearts: Where do we Stand?” Pericytes were first found in the nineteenth century by Rouget. These cells surround capillaries in every organ of the human body, and he indicated the possible use of pericytes as a novel therapeutic avenue in regenerative medicine. He emphasized that pericytes are tissue-specific, multi-functional cells that are capable of treating vascular diseases. Dr Madeddu’s research activity examined the therapeutic effect of pericytes regarding ischemic heart disease, given the ability of pericytes to regenerate and repair heart tissue after myocardial infarction.

Professor Dr Amanda Varnava continued the session on “The Ultimate Goal: Is Gene Therapy in Hypertrophic Cardiomyopathy Yet Possible?” Dr Varnava has an interest in the cardiology of child-bearing period and runs a specialist pregnancy and heart disease service. Hypertrophic cardiomyopathy is the most prevalent congenital heart condition, affecting 1 in 500 of the population with devastating incidences of sudden cardiac death among young people. It is shown that the underlying genetic basis of the disease concerns gene mutation in the gene encoding of the cardiac sarcomere apparatus. A single change in the encoding system may lead to protein degradation and malfunction. Sequentially, sarcomeric dysfunction is inevitable as well as hypertrophy and myocardial fibrosis. Even though no therapeutic options are available to date, she discussed the importance of these molecular targets and suggested new targeted therapies to avoid complications and limit the mortality rate.

Professor Dr Gerasimos Filippatos gave a talk about “Heart Failure Update.” It was shown that sodium-glucose cotransporter 2 inhibitors, as drugs that improve the symptoms of heart failure and improves the left ventricular ejection fraction (LVEF). However, it remains unclear how these drugs benefit heart failure, as he clearly pointed out. Another second-line agent that was found to control heart failure outcomes is the oral soluble guanylate cyclase stimulator vericiguat, which is used for patients who have a reduced LVEF. Concerning inotropes, in patients who suffer from progressed heart failure with reduced ejection fraction, the myosin activator omecamtiv mecarbil can also improve HF outcomes again as he explained. Researchers have focused on the effect of diuretics, as when they are used in combination with other drugs, as they can improve both diuretic response and relieve congestion in hospitalized HF patients. Moreover, as he noted, diabetics and patients with chronic kidney diseases that are given non-steroidal mineralocorticoids in combination with spironolactone and eplerenone can have a positive effect on their cardiovascular and renal function. Prof. Filippatos concluded that in the field of ventricular assist devices, transdermal charging is the new frontier, as it eliminates the need for external leads providing a lower risk of infection and a better quality of life.

Professor Dr Vasso Apostolopoulos spoke on “Vaccines in The New Era: What Have We Learnt in The Last 30 Years?” Recently, her interest has shifted on how chronic diseases, such as cancer, autoimmune disorders, mental health, and infectious diseases, can be treated if approached from an immunologic perspective. The current research on checkpoint markers is shown to lead to apoptotic T cell behavior and immune escape mechanisms in the event of cancer. In the last 5 years, researchers have published numerous information about checkpoint markers as they relate to diseases such as autoimmune disorders, inflammatory disorders, and cancer. Peptide alterations of T cell epitopes with 1–2 amino acid mutations can control immune responses, by downregulating or upregulating feedback. The aim of her research is to reinforce innovative immune modulators/therapeutics/vaccines. Several innovative immune modulators against cancer, autoimmune disorders, and infectious diseases have been successfully established.

Professor Dr Kevin Harrington gave a speech entitled “Is There a Rationale for Combining Radiotherapy and Immunotherapy in Patients with Head and Neck Cancer?” Dr Harrington is a clinical oncologist who specializes in the development of novel treatments concerning head and neck cancer, for which he led multiple phase I, II, and III trials. In CheckMate-141 and KEYNOTE-040 and -048 studies, it was found that these agents, when combined with radiotherapy, are active regarding palliative treatment of relapsed and metastatic diseases. In preclinical studies, it was advised that ICPI therapy should be given simultaneously with RT. This suggestion was generalized into trial designs based on anti-PD1/-PD-L-1 therapy given 1 week before. The results of this study brought negative endpoints, similar to other studies, which also delivered negative outcomes at primary and secondary points, as he pointed out. The presentation greatly focused on the innovation of strategies to enhance the development of combination regimens for patients suffering from locally advanced head and neck cancers.

Ran Nir-Paz presentation entitled “The Enemy of Your Enemy is Your Friend – The Reintroduction of Bacteriophages for Resistant and Persistent Infections.” His study focused on introducing phage therapy for resistant and persistent infections. Recently, the treatment method with bacteriophages for preserving infections has reappeared. The research involved a wide phage band with over 500 identified phages used to discover the most effective lytic phage and to develop treatment schemes. After supplying 15 Israeli patients with intravenous bacteriophages, it was found that 50% of the requests concerned respiratory, skin, and soft tissue infections. The clinical trials mentioned above were further analyzed in his lecture.

